# Exploring the Care Relationship between Grandparents/Older Carers and Children Infected with HIV in South-Western Uganda: Implications for Care for Both the Children and Their Older Carers

**DOI:** 10.3390/ijerph120202120

**Published:** 2015-02-13

**Authors:** Rwamahe Rutakumwa, Flavia Zalwango, Esther Richards, Janet Seeley

**Affiliations:** 1 Medical Research Council, Uganda Virus Research Institute, P.O. Box 49, Entebbe, Uganda; E-Mails: flavia.zalwango@mrcuganda.org (F.Z.); janet.seeley@mrcuganda.org (J.S.); 2 Department of International Public Health, Liverpool School of Tropical Medicine, Pembroke Place, Liverpool L3 5QA, UK; E-Mail: esther.richards@lstmed.ac.uk; 3 Department of Global Health and Development, London School of Hygiene and Tropical Medicine, London WC1E 7HT, UK; E-Mail: janet.seeley@lshtm.ac.uk

**Keywords:** children, older carers, grandparents, reciprocity, care relationship, HIV/AIDS, Uganda, sub-Saharan Africa

## Abstract

The care of children orphaned by HIV/AIDS in sub-Saharan Africa is often undertaken by grandparents, yet little is known about the care relationship between grandparent and grandchild. Our aim was to examine this relationship to understand the needs and responsibilities of both the HIV positive child and older carer and the nature of the relationship, and to assess the implications for care for the children and the older carers. A qualitative study was conducted with 40 purposively sampled children (13–17 years) and their older carers (50 years and above). Participants were recruited from two clinics in south-western Uganda. Up to three semi-structured interviews were held with each participant. Data were analysed using a thematic framework approach. We found that the care relationship was mostly reciprocal: HIV positive children depended on carers for basic and health needs and carers counted on the children for performing tedious household tasks. The relationship was also characterised by challenges, sometimes causing tension between child and carer. We conclude that: (1) interventions targeting HIV positive children need to also address the needs of older carers, and (2) carers and children would benefit from psychosocial support and social protection.

## 1. Introduction

Globally, over 35 million people are living with HIV, of whom 3.2 million are under the age of 15 years [1]. An estimated 2.1 million adolescents (10–19 years) were living with HIV in 2012 in low- and middle-income countries [1]. However, statistical data on young adolescents (10–14 years) are limited and this is likely to impact on the HIV response efforts for this age group [1]. Sub-Saharan Africa remains most severely affected by HIV/AIDS [2]. It has been estimated that of the 190,000 children living with HIV in Uganda, 176,948 are below 15 years [1]. Most HIV infections in children occur during the perinatal period, and have resulted from mother-to-child transmission of HIV. This implies a significant burden of care considering that the children under 15 years constituted the majority (93%) of HIV positive children, and were much more likely to be entirely reliant on a carer compared to their counterparts who were 15 years or over [2].

Over the last three decades, the HIV epidemic has left many families in sub-Saharan Africa severely affected by the loss of family members, who performed crucial roles and shouldered key responsibilities [3,4]. A key feature during this epidemic has been the death of family members, often parents aged 20 to 40 years, resulting in the emergence of many orphaned and sometimes HIV positive children in the years prior to the roll out of anti-retroviral therapy (ART). The burden of care for these children has often been undertaken by older persons, usually grandparents [5,6,7,8,9,10]. 

A number of writers have shown that the care for orphans has ranged from financial support for tuition and other scholastic requirements of older children to day-to-day nurturing care for the younger children [10,11,12,13]. In addition to the challenges in meeting children’s needs, carers faced a variety of health problems usually associated with their advancing age [9,14,15,16]. These older foster carers face multiple challenges which impact negatively on the care-relationship and the kind of care given to the foster child [9].

With ART, there is a growing likelihood that HIV positive children can grow into adulthood even in resource limited countries like Uganda. However, their physical, social and emotional development is determined by several factors and by the nature of the care relationship. Other scholars [17,18] have observed that family and peer level factors affecting children and adolescents with HIV mainly relate to adherence, disclosure and social support. Additionally, it has been found that the style of parenting by older carers, which sometimes contrasts with that of younger foster carers, had various social and health outcomes for the children [9]. For instance, older carers were not comfortable talking to children directly about sex, sexual development and HIV.

HIV positive children are rendered particularly vulnerable, not only because they require very specific biomedical care, but also because they need considerable psychosocial support as they face the challenge of coming to terms with an illness that has been transmitted to them by a parent [19]. Nevertheless, little is known about the social factors mediating access to ART for HIV positive children [20]. Understanding the role older carers play here is critical to ensuring children are able not only to access health care but also education and other social and community support structures [21]. 

The challenges older carers experience are often considerable and can undermine the quality of care for HIV positive children. Furthermore, these challenges may ultimately impact on the nature of older carers’ relationships with the children in their care. It is important therefore to deepen our understanding of the context-specific family processes that facilitate progress in physical, social and cognitive domains of children’s development, and how these processes are influenced by the interplay between parental social support and the changing social, economic and psychological demands placed on the family system [16,22]. 

Our purpose in this paper is to examine the care relationship between older carers and HIV positive children in order to understand the needs of both carer and child, and the possible challenges of caregiving and how these may influence older persons’ relationships with children in their care. We will also identify strategies that might improve care for children and their older carers. 

### Theoretical Orientation

Our theoretical framework draws from the Family Systems Theory (FST). According to the FST, families—or households (a sub-set of a wider family living together in a home or compound), for the purpose of this study—are considered systems because they are made up of interrelated elements/individuals, and have regular interactions among their members who are interdependent on one another [23]. In the context of older carer—child relationship, grandparents and children in their care have different roles and expectations, and needs. But in this particular case, we conceptualise the roles, expectations and needs as revolving around and mediating the provision of social care, that is, both older carer and child have social care duties, and are guided in the provision of such care by roles established normally by the carer, expectations based on these roles, and competing needs of both carer and child. 

Another concept that is relevant for understanding the older carer – child relationship is that of “circular causality” [24]. Circular causality holds that interactions among family or household members trigger a cycle of behavioural responses; that the behaviour of each member influences and is in turn influenced by that of the other members; and that these mutual influences can be positive or negative, and repeatedly reinforce the behaviour of family or household members, thereby sustaining the cycle of responses [25]. We view circular causality, particularly its key feature of reciprocity in terms of mutual behavioural influences, as a useful conceptual tool for understanding the older carer—child relationship. Thus, we propose that when roles are performed as expected and needs met, the behavioural responses of both the older carer and the child are likely to be positive, resulting in an enduring and mutually beneficial relationship. This may not be the case when the older carer and the child are not performing their roles satisfactorily and therefore not meeting each other’s expectations and needs. In this case, the behavioural responses of both parties are likely to be negative, resulting in a deterioration of the relationship. 

We draw on these theoretical perspectives to interpret and discuss our findings, and to recommend strategies for promoting a mutually beneficial care relationship between older persons and HIV positive children. 

## 2. Methods

### 2.1. Sampling

We purposively selected 18 HIV positive children and 22 children whose HIV status was unknown, and their older carers from a clinic run within the General Population Cohort (GPC) of the Medical Research Council/Uganda Virus Research Institute Research Unit on AIDS, a cohort of 20,000 people based in Kalungu District [26] and a Government Health Centre IV, providing paediatric HIV care in the same district. Our working assumption was that those children whose status was unknown were mostly HIV negative.

From each clinic eligible children were selected with the assistance of clinic staff who were asked to select a specified number using stratified random sampling (stratified by gender to ensure that roughly equal numbers of male and female children were included). We recruited 22 children whose HIV status was unknown because we wanted to see if their experiences were different from those of the children who are HIV positive. We obtained a random sample of older carers with children from the GPC. The older carers comprised of seven men and 33 women. These had been drawn in a context where over 2200 older carers of children aged 13–17 years were aged 50 years and above, with a gender distribution of 57% female and 43% male. 

We included children aged 13–17 years and older carers aged 50 years and above. The age of the carers was amended from 60 to 50 years, on realizing that there were more younger older carers in the area than those aged over 60 years. 

### 2.2. Data Collection

Following provision of written informed consent and assent, interviews with participating children were conducted by young interviewers under the age of 25 years, and those with older carers by older interviewers aged over 60 years. Efforts were made to match the participant’s gender with that of their interviewer. Interviews lasted between 45 and 60 min. Pilot interviews provided insights into the participants’ background, HIV/AIDS awareness, and ART treatment (for children on ART), on the basis of which we modified our interview guides for the main study. For the main study, we conducted three separate semi-structured interviews with 40 children and 40 older carers over a period of one year. To ensure sufficient privacy for the children to freely describe their relationship with their carers, interviews were held mostly at school but when they were held at home, sufficient care was taken so that the carer was not in the vicinity of the interview site. In total, a little over 220 interviews were conducted. The team used a topic guide to explore issues about the participant’s background, relationship with carer/child, expectations from carer/child, HIV/AIDS awareness, HIV disclosure, experience with ART and caring practices, among other topics. The interviews were recorded but the interviewers also prepared detailed interview summaries. Data analysis was based on detailed summaries prepared by experienced interviewers and audio recording of participants’ narratives. The rationale for use of summaries was to save the enormous time that would have been spent transcribing all the interviews.

### 2.3. Data Analysis

A framework analysis was done. The team read and reread the detailed interview summaries often referring to the respective audio recordings. Reflecting on the concepts highlighted in our theoretical framework, two overarching categories—the care relationship and caregiving challenges—were created. These categories were then used to organise the themes emerging from the data. With the help of MS Excel, key themes and sub-themes were classified on a matrix, capturing participant narratives accordingly to illustrate each theme. The themes emerging from this analysis included reciprocal care relationship, carers’ poor physical and psychosocial wellbeing, poverty and reliance on cultivation-based livelihoods, inadequate support from family members and other sources, and carer-child tensions. We describe these themes below. Ethical clearance was obtained from the Uganda Virus Research Institute’s Research Ethics Committee and the Uganda National Council for Science and Technology.

## 3. Results 

The findings are based mainly on interviews with 18 HIV positive children and their older carers. However, for comparison purposes we occasionally make reference to perspectives of children whose HIV status was unknown and their older carers. 

### 3.1. The Care Relationship

#### Reciprocal Care Relationship 

Our findings reveal that even in the context of HIV/AIDS where HIV positive children might be viewed simply through the lens of their status as care recipients, the care relationship between older person and child was more complex, demonstrating that carers and children were simultaneously caregivers and care recipients. In 11 of the 18 cases where the older person was providing care to an HIV positive child, both the child and the older carer had needs that they expected to be met within the care relationship. Indeed in these cases, caregiving was reciprocal. 

The HIV positive children looked to their older carers for various forms of support such as providing for basic needs including nutrition, shelter, and education, ensuring that drugs are administered appropriately and on time and that clinic appointments are honoured.

*My grandmother cooks for me tea, food, including vegetables, and if she is not at home, she tells the other children at home to get me vegetables and prepare them. If the food I want to eat is not available at home, my grandmother buys and prepares it for me alone when the others are eating the food that was available.*

(13 year old girl HIV positive)

*I cannot go in the garden before preparing something for him to eat, and we spare something for him from supper for the next morning. Even if it is posho still we spare a piece for him. When he is going to school, I pack something for him to eat. I have sugarcane here and if we are still busy with household work, I tell him to get a piece of sugarcane; I don’t want him to feel hungry. At the clinic I was told that the child had to eat something at any time he liked.*

(64 year female older carer of HIV positive child)

Several children also talked about the care they provided to their older carers.

There are no disadvantages being cared for by an older carer but it is just that my grandmother who would care for me keeps on falling sick and so I take care of her until she recovers and she also does the same.

(13 year old girl HIV positive)

On the other hand, the older carer had a different set of needs, which we categorised as immediate and strategic needs. Immediate needs pertained to instrumental support in executing routine tasks that were necessary for household survival. It is in relation to these needs that the older carer counted on the child for help, particularly in performing household tasks that were too physically demanding for the older carer to perform.

*I am growing old now...Although he has not yet reached that stage of buying me those items, he is the one who fetches water for me now using a bicycle and our well is far from here.*

(56 year female older carer of HIV positive child)

*…he (the child) is the one taking care of me because he fetches the water, brings the feeds for the animals and cooks the food that we eat.*

(60 year female older carer of HIV positive child)

In contrast, older carers’ strategic needs pertained to longer term concerns about household income security and viability, and future care of themselves when the children in their care are no longer available to take care of them. Unlike immediate needs that related to routine instrumental support, strategic needs were psychological and related to the older carers’ wish to overcome the sense of insecurity regarding their future financial and other forms of social care. The carers did not feel confident that they would have anyone to care for them in the future.

*There is a Kiganda (tribal) proverb which says: “better try than never.” Me I take him (HIV positive child) as a cassava stem; it grows wherever you throw it, and in future you can find yourself getting food from it. I am just looking after him (HIV positive child). You never know, God can help and I start gaining from him. When you are a parent, you have hope in your children.*

(56 year female older carer of HIV positive child)

Notably, this view was not restricted to older carers of HIV positive children. One of the carers of children with unknown status had the following to say:

*Every person looks after a child hoping that if the child grows up, he or she might help…when the old person becomes helpless. When they grow up, they have to help me (older person)…Do you think we shall be able to cook food for ourselves in future? We are suffering from different types of illnesses and that is why we have educated this young generation so that they will help us in future. The only worry we have is that what shall we do when they grow up and leave our home? I know God will provide means for us.*

(70 year older carer of child with unknown HIV status).

### 3.2. Caregiving Challenges

In the majority of cases, older persons encountered significant challenges in their caregiving role, which undermined their ability to optimally provide for children in their care. In fact, the practice of children reciprocating care to their older carers may be better understood within the context of these challenges, which included (1) poor physical and psychosocial wellbeing, (2) poverty and reliance on cultivation-based livelihoods, and (3) inadequate support from family members and other sources.

#### 3.2.1. Poor Physical and Psychosocial Wellbeing 

Poor health was one of the major challenges experienced by older persons in their role as carers, affecting 13 of the 18 older carers of HIV positive children. These older carers reported a range of health problems that were mostly associated with their advancing age, including painful backs and limbs, hypertension and diabetes. These health problems limited their ability to execute caring duties within the household, and to access health care for the child.

*I feel lazy, lifeless and with not enough energy. My bones have become weak. I experience pain all over my body; I feel pain in my legs, and the arms feel paralysed.*

(88 year female older carer of HIV positive child)

*(When her HIV drugs are finished and I need to get more from the MRC) I walk on foot yet at the same time I have paining legs. When I walk uphill I feel pain in the chest and I start walking very slowly with pain.... I get very tired.*

(74 year male older carer of HIV positive child)

Another health problem faced by older carers was stress. Stress was usually the result of the carers’ concerns about their poor physical health and the attendant failure to effectively provide for their dependents. But another common source of carers’ stress was general economic hardship—an issue that is explored in more depth in the subsequent section. Depicting the stressful situation she was going through, one carer observed:

*It hurts my feelings because I cannot provide all the necessary things he (HIV positive child) needs. The journey from here to the health centre is very long. When I do not have money I go and kneel before the bodaboda (passenger service motorcycle) riders so that they allow me to pay afterwards*.

(56 year female older carer of HIV positive child)

Similarly, an older carer of a child whose status was unknown had the following to say:

*The father of these children refused to pay school fees for them. He knows I cannot do anything to him since I am an old woman. At times I think that if I was a male person, he would not have refused supporting his children. Now he says that being a woman what I shall do? Had I been…still energetic, he would have feared that I would report him to police.*

(70 year female older carer of child with unknown HIV status)

#### 3.2.2. Poverty and Reliance on Cultivation-Based Livelihoods

Poverty was another major caregiving challenge for older persons, and was reported by 12 of the 18 carers of HIV positive children. In fact, our findings reveal that poverty was closely linked with reliance on insecure small-scale agricultural livelihoods and associated food scarcity problems. A number of older carers were land poor, with others heading landless households. In some cases the cultivated land belonged to the household members. In other cases, where the household did not own land, older carers hired land for cultivation. But some carers, in a determined effort to provide for the children in their care, undertook several concurrent yet physically demanding income generating initiatives. For example, a number of carers undertook cultivation on hired land while also engaging in casual labour:

*I hire a piece of land where we can dig. We first get somewhere to work and get money, which helps to hire another place in turn. Then we can grow maize, beans and potatoes. We also work in the village and get money for commodities like soap, paraffin and other things. When you (interviewer) came I was going somewhere to work. The children do not have anybody else to help them…*

(60 year female older carer of HIV positive child)

Although agriculture provided food for many families, it was vulnerable to serious environmental crises. During the course of our one-year study, participants often complained about food scarcity due to a lack of rainfall in the region, coupled with an inability to purchase food from the market. Indeed older carers of HIV positive children bore the brunt of the food crisis and poverty, as they had to deal with the everyday anxiety about providing an adequate quality and quantity of food for a child on antiretroviral therapy (ART).

*(As a result of drought) getting food is not that easy because maize flour is expensive; so we take porridge (thin maize flour paste) for lunch and make posho (thick maize flour paste) for supper. When we make posho, it is not enough and the boy who is on ART wants more food. He needs a lot of food. I have a big family and so I cannot make posho for two meals each day. There must be enough food for him every day because if he takes three days without enough food, he gets dizzy all the time. That medicine needs a lot of eating and sometimes I get a piece of posho from my share and add it to his. Every time he is looking for something to eat, and so it is a very difficult drug.*

(60 year female older carer of 13 year HIV positive boy)

*Poverty is the greatest challenge that I have, at times I feel like buying him fish but there is no money. They advised us to feed them properly, what I do is to look for greens so that I feed him on them. Because I stay on the roadside, the fishermen pass by here every day, you see him expressing a desire for fish yet I do not have money to buy it. You cannot buy one for him only, you have to buy for the whole family. At times sugar gets finished and I find myself in a miserable situation where I have nothing I can do.*

(64 year female older carer of HIV positive child)

A number of older carers also mentioned the importance of ensuring that the HIV positive child always had something to eat at the point of taking their medication, based on the advice they had received from health workers. However, this was often difficult in a context where the household members did not have food reserves from the previous harvest and the financial means to purchase food from the market.

*You can also examine the situation at our home, we are living in a poor life, we do not have enough food, and so whatever little food I get is what we eat. It is not that I buy things which are special for her; I do not have that money. When I am going very early to work, I just tell her to prepare some tea for herself even if we do not have sugar, because they told her not to take the drugs on an empty stomach.*

(60 year female older carer of 15 year HIV positive girl)

In another case illustrating the hardships many families faced and, as a result, children’s contribution to household income, a carer observed:

*Here at my home each child works for themselves and they support themselves. …Mostly he (HIV positive child) fetches water for other people who pay him 150 to 200 shillings a day, after fetching like five jerry cans. At times when he goes to school with that money he spends it on eats. If you look at his shoes, they are torn and he very much needs shoes.*

(60 year male older carer of HIV positive boy)

Poverty among older persons was a challenge affecting not just those caring for HIV positive children but also carers of children whose status was unknown. This was repeatedly expressed in narratives by both the carers and their children.

One older carer observed:

*My health status is not bad except this food scarcity which is disturbing us. When you go in the village to look for money and you can’t buy food from the money you get only to buy home essentials. So you have to eat what you get, the money I get is not enough for both food and my home essentials. And you can’t rely on casual labouring only, you have to work at home.*

(54 year female older carer of child with unknown HIV status)

This experience was echoed by a child, as noted by the interviewer: about the challenges she has faced being cared for by her grandmother, she told me that she might be in need of Vaseline, clothes and other things yet her grandmother has no money to provide them…She added that she (grandmother) has not been providing what she needs in time. So she is looking for a job during her vacation because she is still wearing the clothes that were bought for her when she was in primary, she has not received any clothes while in secondary ….. (Excerpt from detailed summary of interview with 17 year old girl with unknown HIV status). It should be noted that in Uganda, children especially in the rural areas attend primary school between the ages of six and 15 years, and secondary school between the ages of 13 and 20 years.

#### 3.2.3. Inadequate Support from Family Members and Other Sources

In the context of poor health, poverty and food crises, older carers expected support in the caring role from extended family and other sources. Although the majority reported receiving some form of support from family members, and in other cases, from organisations working in the local area, there were a number of cases where carers and children reported a lack of or inadequate support from these sources. One carer narrated:

*Before I got that (HIV positive) child, we sat in meeting as a family and relatives. It was decided by all to follow his mother’s will that I should keep the child and they would be helping me to meet the needs of the child, but none of them responds.*

(56 year female older carer of HIV positive child)

In another case, an interviewer records a child’s frustration over the lack of family support:

*I asked her (the child) which other relatives can take care of her and she responded that her relatives are there but cannot take care of her because her father died when she was three months and her mother brought her up until 2006 when she was taken by the organization that is taking care of her. She added that their livelihood and the number of children they have cannot enable them take care of her.*

(Excerpt from detailed summary of interview with 15 year old HIV positive girl)

Likewise, concern about family members not providing adequate support towards caring for HIV positive children was sometimes exacerbated in situations where one of the surviving parents got a new spouse who influenced support to the child, as documented in an interview with one child:

*He (the child) told me that he would like to get sufficient support because his father does not support him enough. For instance, “when I go to my father to ask for something I want to eat, he tells me that is not what I want. And in most cases, it is his wife who says that do not give it to him,” he said. His father got another wife and they stay together across the road and it is basically this other wife who mistreats him. When he goes to ask for scholastic materials from his father, he tells him to go back promising to send them but he does not do so. When he asks for school fees of twenty thousand shillings in total, he sends him only ten thousand shillings or five thousand shillings only. He did not pay for his fees the whole of the previous term. He told me he also lacks beddings.*

(Excerpt from detailed summary of interview with 13 year old HIV positive boy).

Perceptions of lack of support for older carers were also expressed by those caring for children whose status was unknown:

*I very much like these children to be educated but I do not have money. I pray to God to see that at least they reach a certain stage. The girl wants to be educated but her father has no responsibility for her.*

(54 year female carer of child with unknown HIV status)

Similarly, most participants perceived a general lack of support from sources other than extended family, with some of the carers expressing pessimism about the prospect of such support.

*In this community of ours, there are no people who have ever come providing support to us, we are always supporting ourselves. I do not even think I can get anything different because of being an old person.*

(64 year female older carer of HIV positive child)

Some of the carers expressed frustration over unfulfilled promises from some individuals or agencies that had initially raised their expectations of much needed support.

*There is nowhere in the area where assistance is given to me as a carer of an HIV positive child. The government even promised to assist older people but has never done so.*

(88 year female older carer of HIV positive child)

This sentiment was also expressed by older carers of children whose status was unknown.

*I have never heard about organisations that offer support here, and there is no one who can help you. Unless someone is your relative and he or she gives you something but there is no other support you can get from anywhere else. Some time back, they came here and registered us, telling us that they were going to offer us mosquito nets but we haven’t yet got them. Another time they called us, the old people, and we went to the trading centre, we waited for the people who were going to register us to get the support for the whole day and they didn’t turn up. Now I have decided not to go back again even if they come.*

(70 year female older carer of child with unknown HIV status)

#### 3.2.4. Tense Relationships

In some cases, the care relationship was characterised by a mutual perception that each was not meeting the other’s expectations and needs. This was exhibited by six of the 18 carer/child dyads. Older carers complained of the challenges of bringing up children, especially adolescents, who would not do as they were told. In one such case, the adolescent would neglect his household tasks by repeatedly staying away from home. These tensions made relationships difficult between older children and their carers:

*I used to advise her (HIV positive child) to behave well but she would not buy my advice and at last she got infected with HIV. She used to move away without my consent and would stay there for some time before coming back home. Whenever I blamed her, she would abuse me and given my age, I just left her for fear that she might attack me and beat me.*

(60 year older carer of HIV positive girl)

Carer-child tensions were also reported by carers of children whose status was unknown:

*The care is not bad but one of the older child gives me a hard time when she comes back late in the evening from school. When I ask her why she becomes annoyed and puts on a tough face.*

(56 year female older carer of child with unknown HIV status)

On the other hand, children expressed perceptions of mistreatment, which indicated that the kind of care they were receiving fell short of their expectations and needs. Some children complained of the harshness of carers:

*I do not quarrel with grandmother like I do with my grandfather (carer) who barks at me whenever I am washing utensils, saying that I should stop. Maybe he wants me to leave the utensils there and the flies fly all over them. Grandmother does not bark at me; she does not quarrel or abuse me.*

(13 year old girl HIV positive)

Other children complained about numerous day-to-day tasks that they were expected to accomplish:

*From digging, we are expected to fetch water and relocate cows from one grazing place to another, which sometimes we fail to accomplish because we are tired…*.

(13 year old girl HIV positive)

Children’s time-consuming domestic workloads sometimes had repercussions beyond the home, as they were often late at school. This was illustrated by school teachers disregarding the children’s seemingly extenuating circumstances, and maintaining a zero tolerance policy on late arrival at classes. Thus, these children were also penalised at school. In one instance, the late arriving child was recurrently denied the opportunity to learn lessons already covered in class, in addition to undergoing corporal punishment, as the above participant told us:

*Then when we get to school late as a result of these (competing) domestic chores, we are also beaten. The teachers just tell us that we should not explain anything regarding the late coming; they beat us and do not repeat for us what we have missed. This happens all the days.*

While this compounded the child’s emotional distress, it also might impact the child’s school performance. 

## 4. Discussion 

Evidence from this study points to the complexity of the care relationship between older carer and HIV positive child, by illustrating that neither the older carer nor the child could be described as purely a caregiver or care recipient. Because this relationship emerged in the context of HIV/AIDS and the child’s seropositive status and need for care, it is easy to perceive it as one in which the care is unidirectional, where the “needy” child is strictly the recipient and the older person the provider of care. However, our findings have shown that HIV positive children in the majority of cases reciprocated care to their older carers. Previous research [27,28,29] has explored reciprocity in parent-child relationships: One of these [28], for example, has described “dyadic reciprocity,” with a focus on meanings, behaviours and social relationships between parent and child. Another study [29] has referred to “dyadic mutuality” as entailing parent-child cooperation, shared positive emotion, and mutual responsiveness. 

Our findings build on those from previous work [9,22] and support the call for recognition of reciprocity of care in older carer—child relationships , lending credence to the argument that it would be inaccurate to view children solely as passive “receivers” in a care relationship [30].

We also observe that because older people play a crucial role in caring for children living with HIV, their needs and general wellbeing are as important as those of the children in their care. This is especially so for those who are suffering from age-related frailty with a heightened need for their own care. We found that a number of older carers, whatever their health status, suffered from stress and anxiety in their role which affected how they looked after the child. This is consistent with other research which has shown that carers of orphaned children, regardless of their age, suffer poor general health and functioning and increased levels of depression, anxiety, and post-traumatic stress [15,31]. On the issue of food scarcity we observe that this is a seasonal problem, although occasionally there are years of more extensive shortages. This lends credence to findings from previous research in the same area which point to this problem as a function of both poverty and environmental crises [32].

Another key finding of our study was the existence of tension within a significant minority (6) of caring dyads, which we attribute to unmet mutual expectations resulting in stress on the part of both carer and child. We observe, for example, that the stress and anxiety that older persons experienced as they provided care to the children in a context of the carer’s physical frailty, and poverty and deprivation might negatively influence the way they related to children in their care. This is consistent with findings from previous studies which point to grandparents’ concerns about child indiscipline and intergenerational disharmony [33], and the stress that young carers experience as a result of shortage of nutritious food, on-an-off education and preoccupation with work [22]. Tension within the context of the older carer-HIV positive child relationship goes against the common perception in the African cultural setting that children are undoubtedly happy and satisfied when they are in the care of their grandparents [34].Viewed within the framework of the Family Systems Theory, tension within older carer/child relationships is the product of unfulfilled roles by some members of the family unit. Indeed as our findings indicated, this tension arose from an older carer’s perception that the child was disobedient and not performing her/his roles as expected, and the child’s perception that the carer was overworking them hence not meeting their caring expectations and needs. 

*Study limitations.* This was a small exploratory study whose findings may not be generalisable to the wider population although the issues raised may be illustrative of wider structural realities. In addition, interviewing adolescents was challenging since they were sometimes reluctant to discuss challenges especially those related to their carers. However, the participatory nature of the techniques used helped to mitigate against these potential pitfalls and ensured that participants felt comfortable to share information.

## 5. Conclusions

The implication of our findings is that programme interventions targeting children living with HIV need to pay attention to the needs of older carers who may struggle to provide emotional and physical support to their children. In particular, there is need for psychosocial support for carers and children, especially where they are living with HIV/AIDS. As well, there is need for social protection initiatives especially for older people in general as they face considerable financial challenges, more so because their livelihoods are largely reliant on subsistence farming that is prone to frequent environmental shocks. These initiatives will benefit those older people caring for children and those who are not.
